# The Multiple Mini Interview for admission to nursing – male perspectives

**DOI:** 10.15694/mep.2018.0000283.1

**Published:** 2018-12-12

**Authors:** Marian Traynor, Iain McGowan, Kathryn Gillespie

**Affiliations:** 1 Queen's University Belfast

**Keywords:** multiple mini interviews, selection, male, values based recruitment, Athena SWAN

## Abstract

This article was migrated. The article was marked as recommended.

**Aims**: The aim of this study was to gain the perspectives of men undergoing recruitment to a nursing degree programme by the process of multiple mini interviews (MMIs).

**Background**: MMIs are used increasingly to select undergraduate students for degree courses, particularly in the healthcare sciences but the impact of MMIs on initiatives to increase gender diversity in these professions is unknown.

**Design**: The study employed a qualitative research approach using a thematic framework of the MMI process.

**Methods**: The study took place between January 2018 - April 2018 and a total of eight students attended for focus groups.

**Results**: Respondents viewed the MMI process as stressful, and also reported that some of the stations created more stress than others, as they were conscious of the gender issues within some of the scenarios. Despite this they also reported the MMI to be a satisfactory selection tool.

**Conclusion**: Participants found the use of MMIs to comprise a valid selection process which, while imperfect and female-dominated, did not unduly disadvantage male candidates. Further research involving multiple nursing schools as well as medical schools is needed to further evaluate the impact of the MMI as a selection tool on male applicants.

## Introduction

Multiple Mini Interviews (MMIs) are being increasingly used in the selection processes for students in a number of professions ( Christensen *et al*., 2018; van der Spuy, Busch and Bidonde, 2016; Andrades *et al*., 2014). They are used to assess a number of professional attributes including communication, empathy, decision making and taking responsibility ( Kelly *et al.,* 2014). Preliminary findings suggest they may be reliable, acceptable, and feasible in the selection of students to the health professions ( Pau *et al*., 2013; Rosenfeld *et al*., 2008; Lemay *et al*., 2007; Perkins *et al*., 2013). Simultaneously, growing evidence indicates that conventional panel interviews are not a robust or consistent method of assessing interpersonal skills for the purposes of admission to medical education ( Andrades *et al*., 2014; Dowell *et al*., 2012; Blouin, Day and Pavlov, 2011; Salvatori, 2001).

However, several authors have also noted that the longitudinal predictive value of MMIs have still to be established ( Gale *et al.,* 2016), while increased scrutiny of nursing education and the nursing profession reiterate the importance of effective recruitment of capable candidates who hold values compatible with the profession.

A number of Schools of Nursing and Midwifery within the UK have now adopted MMIs as a selection tool. However at a time when universities are concerned about ensuring that there is representation from under-represented groups, the perception by school leavers that nursing is considered to be a feminine job, suitable for women, ( Glereana *et al*., 2017) as well as a Nursing and Midwifery Council report that just 10.8% of registered nurses were men (NMC, 2017), it is important to review the MMI with regards to fairness and effectiveness in recruiting under-represented males to the nursing profession and initiatives to increase diversity.

Access to nursing programmes, like all professional courses, is highly competitive. Furthermore, in terms of workforce planning, it is important that departments recruit the right people with the right values who can contribute to patient care and patient safety ( Traynor *et al.,* 2016; Francis, 2013). Recruitment to nursing courses using MMIs has been reported in a relatively small number of papers in comparison to other disciplines. These studies however appear to focus on the development, design, reliability and validity of the MMI process and outcomes ( Patterson *et al*., 2016) with factors such as diversity and implications for widening access less well reported. According to Rees *et al*. (2016) and reported by Curnow (2018), it is however vital that any hidden biases within the MMI system are well understood. Rees *et al.* (2016) suggest that this type of bias occurs when attributes other than those designed to be assessed (e.g. race, age, socio-economic background) affect performance in an assessment.

With regard to bias and MMIs, there is some recent evidence, for example, to suggest that MMIs discriminate against male candidates. Barbour and Sandy (2014) reviewed their experiences over two years running MMIs for entry to a dental school in England. They report that female applicants scored significantly higher than male candidates and were significantly more likely (p=0.016) to receive an offer of a place ( Barbour and Sandy, 2014). These authors do not consider in detail why this may be the case. However, they note existing studies reporting female dental students outperforming their male peers in a number of assessments, and postulate that gender differences in moral reasoning tactics between male and female students may be related to the latter’s stronger scores on the ethics station.

It is noted that studies reporting on MMIs mainly use quantitative methodology and the use of qualitative approaches are scant. This paper will report on a qualitative study carried out in one HEI that sought to explore the MMI experience from the male perspective.

## Methods

Following ethical approval for the study, male nursing students (n= 76) who were successful in recent MMIs were invited to participate. The optimum number of participants that could be accommodated within the fame for the study was four focus groups each with 5/6 participants. Recruitment took place over a three week period during which time the majority of the potential participants were in university classes. Students were advised about the study via email and invited to attend. A total of eight students attended for four focus groups. There were five key questions asked in the focus group ( Table 1). Each focus group lasted circa one hour.

**Table 1.** Focus group question

1. Tell us about your experiences of the MMI process to get into nursing
2. Did you feel, as a male, that any of the scenarios did not allow you to express yourself as fully as you would have liked? Please tell us how and why.
3. What did you like best about the MMI process? Please tell us why?
4. What did you least like about the MMI? Please tell us why?
5. How might the process be improved? Are there any specific changes you would make to help male candidates perform better at the MMI?

## Results/Analysis

Focus groups were recorded and transcribed verbatim. An analysis approach outlined by Miles & Huberman (1994) was applied on data derived from the interactions. NVivo software was used to identify recurring themes, patterns, ideas, shared and diverging experiences.

Miles and Huberman (1994) advocate a three strand approach to data analysis; data reduction, data display and conclusion drawing or verification. They stressed that these three strands are “concurrent flows of activity” (p10).

Data reduction refers to the process of focusing, simplifying, abstracting and transforming the data. Miles and Huberman (1994) argued that it occurs throughout the research process. They contended that the choice of theoretical framework, research questions and research design all influence the data reduction stage of analysis. Data display refers to the “.. organized compressed assembly of information that permits conclusion drawing and action” ( Miles and Huberman, 1994. p11). Conclusion drawing/verification refers to the researcher’s interpretation of the displayed data. It is an iterative process that takes the analyst from the initial meanings- from noting patterns, explanations and propositions - through to gaining a deeper understanding of the phenomenon under exploration, by reflection, iteration and, on occasion, argumentation ( Miles and Huberman, 1994). In order to put into practice this process, the following steps were employed:

1. Focus groups were conducted by one of the team (KG);
2. Recordings of the focus groups were transcribed verbatim;
3. Recordings were listened to again whilst reading the transcripts to ensure accuracy and to check notes on interactions and meaning;
4. Recordings were listened to, and transcripts re-read, on numerous occasions, to aid further understanding of the respondents’ experiences;
5. Responses by each category were coded and synthesised.

Two of the authors (IMcG & KG) coded the transcripts independently of each other. Where disagreements were identified, the third author (MT) facilitated discussion to help reach consensus. KG was unknown to the participants however IMcG was a member of the lecturing team. MT was not known to the students.

### Findings

The experiences of the males taking part in the study were categorised into three areas; the process of multiple mini interviews, specific candidate factors and being a male candidate ( Table 2).

**Table 2.** Male nursing students’ experiences of MMI

Category	Sub-category
Process factors	Anxiety
	Scenarios- Being uncomfortable
	Scenarios- Role play
Candidate Factors	Age
	Previous applications
	Stigma
Being a male candidate	Being in a minority
	Dominance of females in nursing
	Male interviewers

### MMI Process Factors

Anxiety

For all of the participants in this study their only experience of the MMI interview approach had been through their applications to nursing courses. The participants report that, as with all interviews, they experienced a degree of anxiety around the interview process and this was exacerbated by a fear of the unknown. (One participant had one previous experience of the MMI in another nursing school). One participant, who had been part of the first cohort selected by MMI, expressed an additional anxiety about being in a perceived experimental approach to student selection

*“I was very aware that we were the first em, people going to be undertaking it. So it was interesting alone to see, ooh, we’re the guinea pigs here you know.”*(M1)

Being uncomfortable in the scenarios

Participants expressed reservations around the scenarios that required them to either role-play or articulate how they would respond to a distressed and vulnerable woman or child in public areas. They were conscious of how their actions and desire to help, in conjunction with unhelpful stereotypes of men, could be perceived as threatening or inappropriate;

*“I suppose the one about the lady in the shopping mall who was in distress, you know [sighs], the male-female thing is always a, in life, in any shopping centre right now, how would that look even, you know. It’s much more, eh, you would expect a female to be right in there, and a guy to approach with a female in distress, if she’s in distress, you might, you know, that alone, can add more distress, a guy approaching a total stranger, with a beard [laughs]”*(M4)

This participant drew on a traditionally masculine physical characteristic (a beard) to highlight his concern that his contribution in a real-life caring scenario may be lessened by the recipient’s immediate and superficial potential perceptions of him *as a man* sitting in contrast with the caring/helping role.

Using role-play

Participants were mixed in their views about the use of role-play in the MMI process. It was recognised that role-play circumvented a reliance on “learned” answers and instead allowed candidates to display their abilities in realistic scenarios:

*“I actually quite enjoyed it, because it gave you the opportunity to, as .. said, go in and show your life experience and show your willingness to care for people, your willingness to look after people. And, instead of sitting in front of an interview panel and rhyming off what you’ve learnt.”* (M6)

Conversely, some participants expressed the idea that role-play added an additional complexity and that it might be easier to explain intended actions rather than act these out:

*“There’s a lot of acting ones, you felt like you were repeating yourself a lot, em, and if there was a silence you felt like, “aw what was I gonna do?” But in the ones where you were explaining, if you had a silence, like, you weren’t judged like, you were just trying to think and if you brought up something else then that was alright.”* (M2)

Where role-play was used, participants felt that the actors could have been more responsive to what was being said and done in order to accommodate candidates’ responses to scenario:

*“It felt like they had a script and things that you would have said in real life could have helped but they were looking for you to say a certain thing and if you didn’t say that certain thing they wouldn’t progress with the conversation then.”* (M3)

Such inflexibility was perceived to precipitate increased stress and self-doubt during that MMI station.

### Candidate Factors

While most participants reported at least some experience of being conscious of their gender as an influential factor during the MMI process, for some this was not as significant as other considerations, such as those relating to age or experience. For example, one participant expressed concern that his young age might have represented a lack of life experience which he perceived might constitute a more substantial obstacle to success than his gender.

*“I’m thinking, what have I got over them. They’ve got so many years of experience.”*(M1)

Another participant considered the significance of experience from a different perspective, comparing his current confidence with how he perceived himself at a younger age. This increased confidence was mentioned by a number of participants, and was seen not only to have improved their MMI performance, but to have been instrumental in their decision to apply to the course. Participants described how such a decision would not have been possible in their youth because of attributing more weight to others’ opinions about them, and having not yet developed the courage to undertake an atypical career path.

The number of times a candidate had applied to nursing/experienced MMIs made a difference to their perceptions of the second experience of the MMIs:

*“I was thinking about how I felt going straight from school and I was just thinking of the advantage I have over these people. And I just felt ten times more confident going in than the first time.”* (M6)

The Specific Impact of Being Male During the MMIs

Participants in the study reported being very aware of their gender both during the selection process and also in seeking to enter a profession where the majority of staff are female.

Being in the minority, or as one participant described,

*“being surrounded by a sea of girls*” (M2)

during the introduction and debriefing sessions of the MMI process appears to have increased anxiety in some of the participants. As such, some participants articulated that they felt that consideration should be given to ensuring that there is a visible and meaningful number of men during each iteration of the interviews

“.. *more males together. I think that calming effect on each other would help improve the process for males* (M6).

Similarly, being interviewed by male staff appeared to offer some comfort to the study participants, due to the assumption of shared male experiences and perceptions

*“As soon as you walk in, he shook your hand [..] I know everybody does it but, it’s, you know, whenever you’re, you know, males greet each other, it’s the manly handshake.” (*M3).

Familiar behaviours such as the handshake were reported as helping male candidates to feel less out of place during the MMI process.

Awareness of female dominance in the nursing profession generally played on the mind of study participants, as did their own experiences prior to applying for nursing courses. One participant recalled feeling a need to prove himself as a male nurse amongst his female peers

*“I think as a male you want to show people that you can, ah you can, as ... said again, you’re going into a field with, ah, dominant of females, so you want to prove yourself as a male*” (M2).

Another related experiences of strong judgement by his local community as a result of his career choice,

*“I’d be very much rural and you’re sort of expected to take a trade after school and that sort of thing. And I’m sort of the black sheep of, not just our family, of our twenty-mile radius, locality, probably, you know*” (M6).

Despite the reservations candidates did find the experience positive:

*“.but I quite enjoyed the process, sounds weird but when I look back on it now, the questions, the scenarios, and how you deal with them, shows quite a lot about yourself and I quite enjoyed it”* (M6)

*“I thought the MMI process itself was okay; I had no issues with it..”* (M4)

*“I don’t think it was just because I was a, I was a male. I think they (stations) were just hard, some of them were just hard in general” (*M1)

## Discussion

The aim of this study was to investigate the experiences of men selected for a nursing degree course by the process of MMIs. Not surprisingly the respondents viewed the interview process as stressful, with the awareness that the interview style was new, compounding their stress levels. They also reported that some of the stations created more stress than others as they were conscious of the gender issues within some of the scenarios, for example, a male offering assistance to a female in distress. Therefore this needs to be taken into account when planning stations for MMIs. Although it is good practice for stations to be subject to external peer review, we recommend that a reference to the station as having potential issues for male applicants should be included in the peer review check list.

Specific actions to counter disadvantage may include writing stations which enable male candidates to demonstrate their capabilities without highlighting how differently “caring” actions may be perceived if performed by a male outside of a nursing context. Failing to take into account participants’ expressed anxieties regarding how their actions may be interpreted may lead to further stress during the MMI process. Further, factors such as decreased comfort and increased self-censorship may directly impact on performance and scoring, in a context where being able to display one’s own inner values and inter-personal skills, is a key element of assessment.

The respondents also made reference to the simulated patients/role players, suggesting that they could have engaged more with the interviewee at the stations. The lack of engagement by the role players had been a deliberate action implemented by the MMI working group in an attempt to standardize the stations. This has since been reviewed and the simulated patients/role players have been provided with a more detailed context about the station. This then gives simulated patients more liberty to converse with the applicant, and helps to avoid what participants perceived as frustrating obstacles to progress, should their valid attempts to engage at each station not sufficiently follow a scenario, which they perceived to be highly scripted and restrictive.

Despite the small sample size in this study there was no inference amongst respondents that they experienced any bias in relation to gender and MMIs and instead were more mindful of their years of experience and how this gave them more confidence. This supports findings by Jerant *et al*. (2015), who reported that older applicants performed better in MMIs. Better MMI scores for older applicants was also reported in a more recent study by Traynor *et al.* (2017), who found that the MMI had a tendency to favour the mature candidate and reported that for every 10 plus years of age, candidates’ MMI scores improved by 5 points. It should however be noted that this study was carried out on a group of first year students who had been successful on the traditional interview format and were already enrolled on a nursing course. The fact that 92% of the participants were female also limits the generalizability of the findings.

The results of this study support the findings of Uijtdehaage, Doyle and Parker, (2011) and O’Brien *et al.* (2011) who did not find gender bias. However, a study by Jerant *et al.* (2012) reported a positive correlation between MMI score and female sex and a study in the University of Coventry ( Humphrey *et al.,* 2008), that assessed candidates’ and interviewers’ perceptions of the MMI, found that interviewers felt the interviews were fair to candidates and were not unfair to male candidates.

Furthermore, a study by Ross *et al*. (2017) found that on the MMI scores of 526 applicants to medical school at the University of Calgary, female students were rated higher than the male applicants (p<0.001), even after controlling for confounding variables such as applicant age etc. The paper offers two possible explanations for this; the first is that women are more likely to demonstrate the attributes that the MMI is intended to capture. For example, when compared with men, the women typically demonstrated better communication skills and were rated better on certain aspects of critical thinking and ethical decision making. The second explanation offered by Ross *et al. (2017)* is that women are rated higher on the MMI because interviewers expect them to be better at communication, critical thinking and ethical decision making. The second explanation is an obvious cause for concern as it suggests interviewer bias towards female applicants.

Another more general but poignant point in relation to men in nursing and applicable to Higher Education and men per se, was raised by one respondent in this study who described an expectation that men from a rural background were expected to take a trade after school. The fact that he was not only applying to university but also applying to a female dominated profession, made him “.. *the black sheep of, not just our family, of our twenty-mile radius, locality..”.* Such experiences represent a clear barrier to participation, especially for young men who have not yet developed sufficient confidence (as described by participants in this study) to follow career routes which have historically been more associated with women.This HEI like many HEIs has identified widening participation as a priority and this type of comment reflects the extent of the work that has yet to be undertaken in this important area.

Simply being a male participant in MMIs for nursing (a female dominated profession) immediately creates a disparity. Consequently, the potential for MMIs to disfavour male applicants is, warrant of monitoring ( Jerant *et al*., 2015). Although respondents from this study did not refer to men being disadvantaged by the MMI, they were nevertheless concerned about the dominance of the number of females that, according to one respondent precipitated a perceived pressure, to “.. *prove yourself as a male”.*

A further reference to the underrepresentation of men applying to nursing and indeed underrepresentation in the nursing profession generally was captured by one respondent who valued a male presence on the day of the MMIs. Specifically, he referred to being greeted by a man and the importance of the *“.manly handshake..”.* As a way of increasing the male presence and increasing opportunities for male collegiality, there was a suggestion by one respondent that all males should be invited to interview on the same day. This is something that can be considered, with the understanding that if it leads to an improved experience for what is a minority group, it is worth pursuing. This suggestion is not without precedence as there currently exists, for example, women only mathematics competitions, which helps to improve women’s performance ( The Guardian, 2015).

Given that women are becoming the dominant gender in other professions such as medicine, and the strong influence of performance at MMIs on, for example, ultimate acceptance to medical school, the findings of this study are worthy of noting across disciplines particularly for those charged with writing MMIs and assessing stations.

### Limitations

This study was about male candidates’ perception of the MMIs and therefore has limitations. Although the candidates interviewed agreed that the MMI was most likely a better selection process than the traditional interview, they were conscious of the huge disparity between male and female applicants, and much like the findings of Humphrey *et al.* (2008) their agreement reflects only opinion and does not imply fact.

This study included only current male undergraduate nursing students, who had already received and accepted a place following a successful MMI process. Participants were able to discuss their perceptions of other male candidates they had encountered during the MMI process who did not secure a place; however, not including the latter in their own right limits this study.

Furthermore, the data was gathered from one School of Nursing and given the low number of respondents it is difficult to say how widely the findings could be generalised.

## Conclusion

This study sought to fill the gap in what is recognised as an under-researched group i.e. the views of men undertaking MMIs for application to nursing. Participants found the use of MMIs to comprise a valid selection process which, while imperfect and female-dominated, did not unduly disadvantage male candidates. Further research involving multiple nursing schools and ideally medical schools, is needed to further evaluate the impact of the MMI as a selection tool on male applicants.

## Take Home Messages

• Writing MMI stations: consideration must be given to potential gender issues
• Examing at MMI stations: consideration must be given to potential unconscious bias towards female applicants
• Context of scenarios for simulated patients: provide adequate information so that there is sufficient material for SP to converse with the applicant
• Further research involving multiple nursing schools and ideally medical schools, is needed to further evaluate the impact of the MMI as a selection tool on male applicants
• Give consideration to MMI gender specific circuits

## Notes On Contributors

**Dr Marian Traynor** is Associate Dean of Education for the Faculty of Medicine, Health and Life Sciences, Queen’s University Belfast. She holds a range of academic and professional portfolios and has published and presented her work nationally and internationally. Dr Traynor has a particular interest in values based recruitment and recently introduced Multiple Mini Interviews (MMIs) to the School of Nursing and Midwifery at Queen’s.

**Dr Kathryn Gillespie** recently completed her PhD at the School of Nursing and Midwifery, QUB, looking at relationship and sexuality education for young men. Kathryn is currently acting as a postdoc researcher supporting the UK wide cluster randomised controlled trial of an RSE intervention entitled ‘If I were Jack’ in schools.

**Mr Iain McGowan** is a mental health academic and clinician with a particular interest in suicide assessment and prevention, trauma and its related presentations, the provision of care for heroin users, infant mental health and the education of mental health nursing students. He is currently a Lecturer in Education at Queens University, Belfast and a former Editor in Chief of the editorial board of the International Journal of Clinical Psychiatry. Iain recently completed a Post- Graduate Diploma in Applied Health Economics.
