# Supplementary material for: The Multiple Mini Interview for admission to nursing – male perspectives
Source: MedEdPublish (2016). 2018 Dec 12;7:283. [Version 1] doi: 10.15694/mep.2018.0000283.1 (PMC10711962; doi:10.15694/mep.2018.0000283.1)
Supplement: Peer review history

# Reviewer report: Reviewer response for version 1

**DOI:** 10.21956/mep.19719.r29519

**Published:** 2019-01-05

**Authors:** Mohammed Osman

**Affiliations:** 1 General orgainization for teaching hospitals

This review has been migrated. The reviewer awarded 4 stars out of 5 The manuscript is very interesting and adds a lot to our knowledge. Also, I find the findings and (take home messages) are very important. Few points might improve the quality of this interesting research including to improve the sample size, to conduct the trial in more than one institute and to give more data about the candidates; Was this their first employment,....etc. Better inclusion criteria is my main advice. Thanks

Reviewer Expertise:

NA

No decision status is available

# Reviewer report: Reviewer response for version 1

**DOI:** 10.21956/mep.19719.r29522

**Published:** 2018-12-25

**Authors:** Ken Masters

**Affiliations:** 1 Sultan Qaboos University

This review has been migrated. The reviewer awarded 2 stars out of 5 The paper examines males’ experience of MMIs for a nursing degree programme. The topic is important, and, as the authors note, as the gender balance of other health professional paths change, will become increasingly important there also. In addition, the decision to use a qualitative study is commendable, as first explorations into a topic are usually well-served by qualitative studies.A serious issue arises, however, in the execution of the study, primarily as a result of the small sample size. Although the authors do note in their limitations that there was a “low number of respondents”, the composition of the focus groups is not clear, and the small sample size combines with the methodology to undermine the study, and, therefore, the results: The authors tell us that eight students attended four focus groups, but they do not say how many were in each group. At most, there would have been two, which is not a group, but a pair, and immediately the dynamics of the “group” are altered, and the strength of a focus group is lost. If there had been any more than two in any group, then at least one “group” would have been a single student. This is even worse, as it is an interview, and not a focus group in any sense of the word.It was at this stage, before interaction with the students was started, when the authors realized that they had such a low number of participants, that they should have made a fundamental change to the study. Options would be:• Set it up as a small pilot study, and as an entrance to a larger research study (and part of the pilot would be to try to determine why so many of the other students did not wish to participate, with a view to solving that problem.)• Change the nature of the interactions to individual interviews, and change the theoretical framework to something like Grounded Theory, or any other qualitative framework that emphasizes simultaneous theory development with data gathering, and also has limitations set by saturation. (There are cases in qualitative research where sample size is less important than in quantitative research, but, in those cases, the researchers use other controlling factors, such as reaching saturation point. No such control was performed in this case.)• Combine these two bullets.• Abandon the study completely (although this should be a last resort only).So, although there are some interesting insights offered by the students in this study, the small size combined with the methodology means that it cannot be viewed as a research article, and rather only as an interesting small pilot study. I would argue that it would be rather dangerous and irresponsible for anyone to draw hard conclusions and make changes to their MMIs based on the results of this pilot, which appears to be rather prematurely published: the appropriate path forward would surely be to design and implement a broader and larger research study.

Reviewer Expertise:

NA

No decision status is available

# Reviewer report: Reviewer response for version 1

**DOI:** 10.21956/mep.19719.r29521

**Published:** 2018-12-13

**Authors:** Barbara Griffin

**Affiliations:** 1 Macquarie University

This review has been migrated. The reviewer awarded 3 stars out of 5 The performance of males on admissions interviews has become very topical and this study gives some good insights. In particular, it demonstrates how MMI developers must think through the implications of scenarios so they are not providing an biased assessment (e.g. creating a situation where there are different social norms for males and females, such as described with one of the role plays). I would like to see this qualitative data extended by looking at the associated quantitative data (e.g., did males get lower scores on the role playing stations?). Importantly, it is difficult to draw too many conclusions when the study does not compare issues raised by males with those that might be raised by females - there may not be as many differences as one might expect.

Reviewer Expertise:

NA

No decision status is available

# Reviewer report: Reviewer response for version 1

**DOI:** 10.21956/mep.19719.r29520

**Published:** 2018-12-12

**Authors:** Linda Nield

**Affiliations:** 1 West Virginia University School of Medicine

This review has been migrated. The reviewer awarded 4 stars out of 5 This topic is interesting and novel. It would be most interesting to include those applicants who were not successful, as the authors have acknowledged this limitation to the work. Three minor suggested edits: 1. I may have missed this in the text, but the abbreviation "HEI" needs to be explained 2. In "Take Home Messages" number 3, the abbreviation "SP" needs to be explained 3. In "Take Home Messages" number 4, the comma needs to be removed.

Reviewer Expertise:

NA

No decision status is available

# Reviewer report: Reviewer response for version 1

**DOI:** 10.21956/mep.19719.r29523

**Published:** 2018-12-12

**Authors:** Trevor Gibbs

**Affiliations:** 1 AMEE

This review has been migrated. The reviewer awarded 4 stars out of 5 Thank you for asking me to review this paper. I enjoyed reading it and I felt that it began to make faculty think about another aspect of the MMIs, the research on which is still on-going. The authors presented their piece of research very clearly and pointed out the strong limitations to ant findings they discussed. I do think that this paper could be of use to all involved in MMIs, although I feel that it only opens the door slightly to a rather bigger issue- all research needs to start somewhere. I was left with some doubt in my mind about how males would feel about standard interviews for a job / vocation that is so strongly female-dominated. Is it the MMI that makes these feelings as described?

Reviewer Expertise:

NA

No decision status is available
